# *Schisandra chinensis* Fructus and its active metabolites for the treatment of metabolic dysfunction-associated steatotic liver disease

**DOI:** 10.3389/fmed.2026.1743905

**Published:** 2026-05-13

**Authors:** Li Jun Wang, Ping Zhang, Gui Li Wang, Shu Cheng Chen, Wei You Cao, Ji Chun Han, Jian Guang Sun, Jian Chao Feng, Yang Zheng

**Affiliations:** 1 Department of Traditional Chinese Medicine, Shandong Medical and Pharmaceutical University Yantai, China; 2 Nanxiang Hospital, Shanghai, China; 3 School of Nursing, The Hong Kong Polytechnic University, Kowloon, Hong Kong SAR, China; 4 The First Clinical Medical College of Shandong University of Traditional Chinese Medicine, Jinan, China; 5 Department of Infectious Diseases, Shanxi Provincial Hospital of Integrated Traditional Chinese and Western Medicine, Taiyuan, China; 6 Department of Geriatrics, Zibo Hospital of Integrated Traditional Chinese and Western Medicine, Zibo, China

**Keywords:** active metabolites, metabolic dysfunction-associated steatotic liver disease, pharmacological effects, research progress, *Schisandra chinensis*

## Abstract

*Schisandra chinensis* Fructus (SCF) is a traditional Chinese herbal medicine with both medicinal and culinary uses. Its main active ingredients, including lignans, triterpenoids, volatile oils, and organic acids, have been utilized to treat liver diseases. SCF extract exhibits pharmacological activities such as protecting hepatocytes, reducing hepatic fat accumulation, alleviating insulin resistance, and mitigating oxidative stress, thus holding promising application prospects in alleviating metabolic dysfunction-associated steatotic liver disease (MASLD). The aim of our review is to summarize and organize recent literature regarding the active metabolites and pharmacology of SCF, so as to clarify the pharmacological mechanism of this herbal medicine and provide a reference for the research, development, and utilization of SCF, its compound preparations, and related health foods. Literature selection was performed using relevant databases in traditional Chinese medicine and biomedical sciences, based on the ethnopharmacological uses (medicinal and culinary) of SCF. Studies investigating the roles and mechanisms of SCF’s active metabolites in alleviating MASLD were primarily chosen. SCF contains multiple active metabolites, among which lignans, triterpenoids, volatile oils, and organic acids play crucial roles in alleviating MASLD. These metabolites exert pharmacological effects by protecting hepatocytes from damage, regulating hepatic lipid metabolism, improving insulin sensitivity, and resisting oxidative stress, collectively contributing to the mitigation of MASLD. However, systematic exploration of the integrated mechanisms involving these metabolites remains necessary. The active metabolites of SCF possess diverse pharmacological activities in alleviating MASLD. Clarifying their underlying mechanisms is beneficial for promoting further research, development, and application of SCF in medicine and health foods, providing a solid basis for related drug development and functional food utilization.

## 1 Introduction

*Schisandra chinensis* (Turcz.) Baill. Fructus (SCF) is the dried mature fruit of *S. chinensis*, a plant from the magnolia family. SCF encompasses all five flavors: sweet, sour, pungent, bitter, and salty. A well-known ingredient in Traditional Chinese Medicine (TCM), SCF has a millennia-long medicinal history. The earliest record is the *Divine Farmer’s Materia Medica*, which states that it “benefits qi, alleviates cough and shortness of breath, treats weakness and emaciation, replenishes deficiencies, strengthens the kidneys, and benefits male essence.” SCF has relatively mild medicinal properties and is associated with the lung, heart, and kidney meridians. Its effects include astringing and solidifying, benefiting qi and generating fluids, and nourishing the kidneys and calming the heart. It is used clinically to treat chronic cough, weakness-induced asthma, nocturnal enuresis, frequent urination, internal heat and thirst, spontaneous sweating and night sweats, persistent diarrhea, palpitations, and insomnia. The *Chinese Pharmacopeia* lists 100 formulated preparations containing SCF, which are often used in combination with yin-nourishing, qi-replenishing, and diuretic and dampness-draining herbal medicine (1). For example, when SCF is used in combination with dwarf lilyturf tuber, *Rehmannia glutinosa*, and *Poria cocos*, the therapeutic effects are enhanced.

SCF is a widely utilized medicinal plant in modern therapeutics. Its fruits have been extensively employed in traditional medicine systems across China and Russia for treating diverse disorders (2). In the early 1960s, SCF was formally recognized as an adaptogen by Soviet medical authorities, primarily stemming from extensive pharmacological and clinical research conducted by Russian scientists during the preceding two decades. This status is evidenced by its inclusion in the latest editions of the State Pharmacopeia of the Union of Soviet Socialist Republics (USSR) and the National Register of Medicinal Products (3). Introductions to SCF can be found in the pharmacopeias of Japan (4), South Korea (5), the United States, and Russia (6, 7), and in the *International Pharmacopeia* published by the World Health Organization (8).

**Figure 1:**
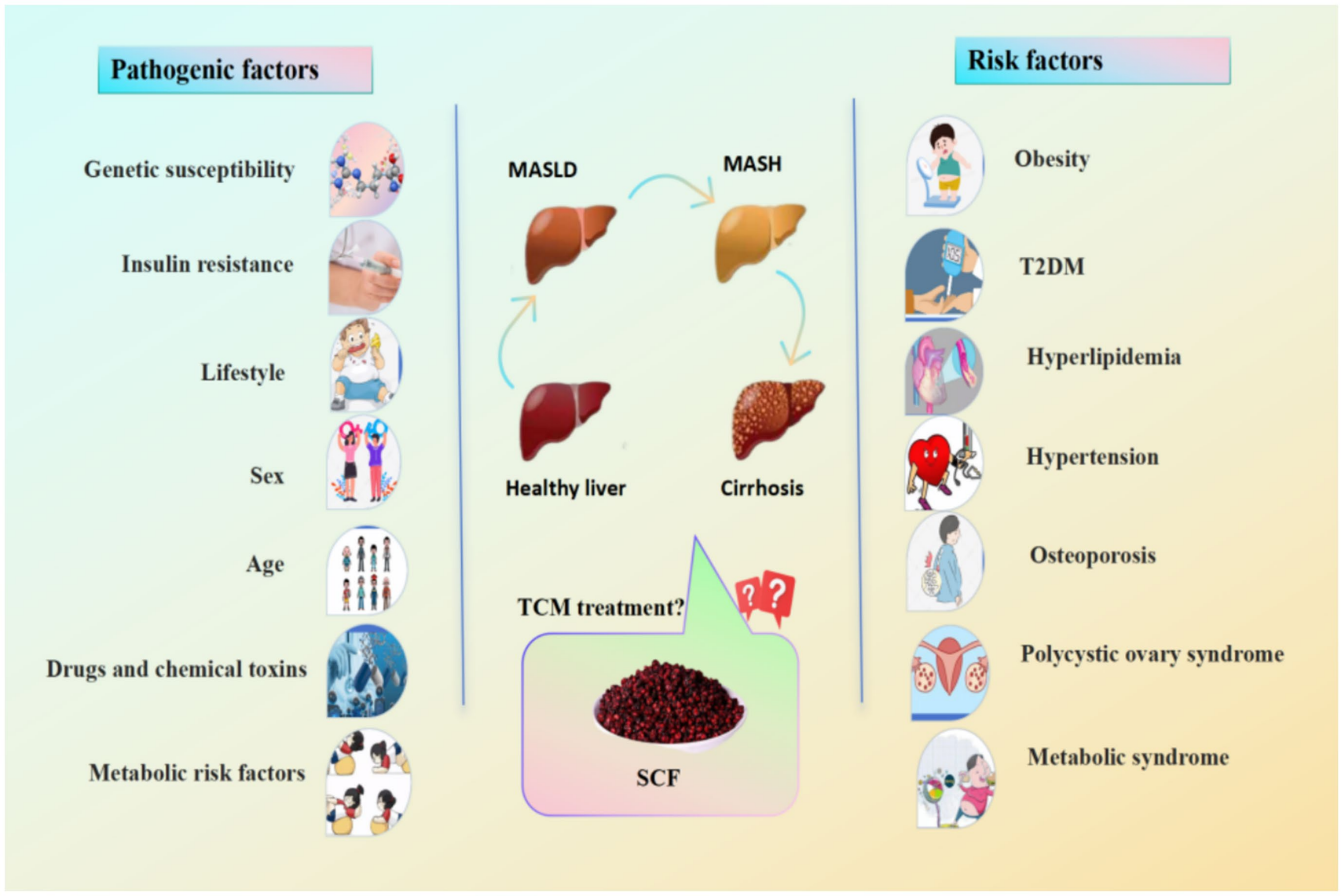
Pathogenic and risk factors for the development of MASLD. The main pathogenic factors for MASLD are genetic susceptibility, insulin resistance, lifestyle, sex, age, drugs and chemical toxins, and metabolic risk factors. MASLD is highly likely to progress to metabolic dysfunction-associated steohepatitis (MASH) and cirrhosis. Other risk factors include obesity, type 2 diabetes mellitus (T2DM), hyperlipidemia, hypertension, osteoporosis, polycystic ovary syndrome, and metabolic syndrome.

Metabolic dysfunction-associated steatotic liver disease (MASLD), previously known as non-alcoholic fatty liver disease (NAFLD), is a chronic disease characterized by lipid accumulation and fatty degeneration in liver cells, after excluding alcohol and other clear causes of liver damage. As MASLD progresses, persistent fat accumulation can induce oxidative stress (OS) and inflammatory responses, leading to metabolic dysfunction-associated steatohepatitis, which may ultimately result in liver fibrosis, cirrhosis, and in severe cases, liver cancer. The pathogenesis of MASLD is complex, involving lipotoxicity, OS, insulin resistance (IR), and changes in the gut microbiota (GM) (9). Due to high-calorie diets and sedentary lifestyles, MASLD is a major cause of chronic liver disease, with annually increasing incidence (10). A recent epidemiological meta-analysis indicated that the global prevalence of MASLD is approximately 25% (11). South America and the Middle East have the highest prevalence, followed by Asia, while Africa has the lowest (12). By 2030, the total number of cases of MASLD is predicted to increase by 18.3%, with the number of cases related to advanced liver disease and liver-related mortality doubling (13), posing a significant challenge to health. This increasing public health issue will exacerbate the global healthcare and economic burdens. The pathogenesis of MASLD is driven by complex interactions among metabolic dysfunction, lipotoxicity, and genetic susceptibility (see Figure 1), which schematically illustrates the multifactorial etiology and key risk factors including obesity, insulin resistance, and hyperlipidemia.

As a metabolic syndrome, MASLD is usually asymptomatic, but it is closely associated with obesity, type 2 diabetes (T2D), hypertension, dyslipidemia, and polycystic ovary syndrome (14, 15) A 2016 study found that osteoporosis, colorectal cancer, sleep apnea, psoriasis, endocrine disease, and pancreaticoduodenectomy are also risk factors for MASLD (16). Moreover, the severity of MASLD is closely related to genes, environment, and behavior (17, 18). Obese people are more likely to develop fatty liver disease than lean people. Bellentani et al. (19) showed that obese individuals with a body-mass index (BMI) ≥ 30 kg/m^2^ have a MASLD prevalence of 94%, while that for overweight individuals with a BMI ≥ 25 kg/m^2^ is 67%. The risk of MASLD extends to adolescent and child groups (20). In the United States, approximately 9.6% of children and adolescents aged 2–19 years have fatty liver disease, with this proportion rising sharply to 38% among those with obesity (21). Data from the United Kingdom indicate that nearly one-fifth of adolescents develop fibrotic progression by age 24, while the overall pediatric prevalence reaches 1 in 40 (22). Asian studies report an overall fatty liver prevalence of 5.5% in children, escalating to 50.1% among obese pediatric populations (23). Globally, the estimated prevalence of fatty liver disease in the general pediatric population ranges between 3 and 10% (24).

MASLD is mainly treated with drugs for obesity, hypertension, blood lipid disorders, diabetes, and other diseases, which generally have poor effectiveness and cause liver toxicity; there are currently no MASLD-specific drugs (25). Meanwhile, interventions mainly focus on lifestyle changes such as diet adjustment and exercise enhancement, with unsatisfactory results due to poor compliance, mild effects, and high likelihood of rebound (26). Therefore, natural products with high safety and targeted action have become the focus of drug development.

SCF is not only widely used in TCM but has also received increasing attention in pharmacological research. Compared with other drugs for fatty liver, SCF has advantages such as a multiplicity of targets, pathways, and mechanisms, and mild adverse reactions. In recent years, it has become a candidate drug and research hotspot for the prevention and treatment of MASLD. Evidence increasingly shows that SCF alleviates the symptoms of MASLD through pathways such as the farnesoid X receptor (FXR)/fibroblast growth factor 15 (FGF 15) (27), FXR/Takeda G-protein-coupled receptor 5 (TGR5) (28), and glycogen synthase kinase-3β (GSK3β) signaling pathways. A network pharmacology analysis based on 50 chemical metabolites in SCF identified 246 potential targets for treating MASLD, suggesting that the amelioration of MASLD by SCF is related to pathways associated with lipid metabolism and OS (29). SCF demonstrates significant therapeutic advantages and potential for treating fatty liver.

## 2 Literature and data search methodology

Systematic reviews remain scarce, with most existing reports focusing on the pharmacology and mechanisms of specific chemical classes in SCF. Consequently, this article comprehensively summarizes and reviews recent research from PubMed, Web of Science, Cochrane, CNKI, and Scopus concerning the alleviating effects and underlying mechanisms of SCF against fatty liver. The literature search was conducted for studies published from 2000 to the present concerning.

SCF and its active metabolites in treating fatty liver disease. Search terms included “*Schisandra chinensis* (Turcz.) Baill.,” “*S. chinensis*,” “Wuweizi,” “SCF,” “SCF metabolites,” combined with “Lignans,” “Triterpenes,” “Schisandrol,” “Volatile Oils,” “Polysaccharides,” “Organic acids,” and “Flavonoids.” These were further filtered using terms related to fatty liver disease: “fatty liver,” “non-alcoholic fatty liver disease”, “NAFLD”, “fatty liver hepatitis”, “metabolic associated fatty liver disease”, “MAFLD”, “metabolic dysfunction-associated steatotic liver disease”, and “MASLD.” This work provides theoretical support and a reference for research on TCM approaches to the prevention and treatment of liver diseases, and important scientific evidence for developing safe, effective drugs for treating fatty liver.

## 3 Chemical metabolites

SCF contains a variety of bioactive metabolites, such as lignans (30), terpenes (31), polysaccharides (32), volatile oils (33), and organic acids (34). SCF has liver-protective effects (35, 36), regulates glucose and lipid metabolism (37), exhibits antioxidant properties (38, 39), and provides neuroprotective (40), anti-aging (41), and anti-cancer effects (42). A molecular docking study found that 44 active metabolites of SCF docked with protein targets related to liver diseases, including fatty liver, viral hepatitis, liver fibrosis, and liver cancer. Among these metabolites, schisandrin, schisandrol B, kadsurin, gomisin A, gomisin G, and angeloylgomisin may target 21 intracellular proteins associated with liver diseases, particularly fatty liver disease (43).

SCF contains a diverse array of bioactive metabolites spanning multiple chemical classes (see Table 1), which catalogs 18 representative compounds including biphenyl cyclooctene lignans, triterpenoids, and cyclohexane derivatives, with their respective molecular formulas, molecular weights, and bibliographic references.” Among the 18 chemical metabolites listed in Table 1, the biphenyl cyclooctene lignans represent the most extensively studied class of bioactive compounds in the context of MASLD. These lignans, including schisandrin B (Sch B), schisandrin A (Sch A), and deoxyschisandrin (DS), have been demonstrated to ameliorate MASLD through multiple mechanisms: activation of PPAR-α to promote fatty acid (FA) oxidation, inhibition of the LXR-α/sterol regulatory element-binding protein 1c (SREBP-1c)/FAS pathway to reduce *de novo* lipogenesis, activation of the AMPK/mTOR autophagy pathway to decrease hepatic lipid accumulation, and suppression of pro-inflammatory cytokines to attenuate hepatic inflammation. Additionally, triterpenoids and cyclohexane derivatives may contribute to the hepatoprotective effects, although their specific roles in MASLD require further investigation.

**Table 1:** Chemical metabolites of SCF.

No.	Compound name	Category	Molecular formula	Relative molecular weight	References
1	Schisanchinin A	Biphenyl cyclooctene type	C_30_H_30_O_8_	518.56	(144)
2	Schisandroside D	Biphenyl cyclooctene type	C_33_H_42_O_14_	662.68	(145)
3	3,7-dihydroxy-1,2,13,14-tetramethoxydibenzo-cyclooctadiene-12-O-β-D-glucopyranoside	Biphenyl cyclooctene type	C_28_H_38_O_12_	566.60	(146)
4	Schisandroside E	Biphenyl cyclooctene type	C_32_H_44_O_13_	636.69	(147)
5	(R-biar)-12-angeloyloxy-6,7,8,9-tetrahydro-1,2,3,13,14 pentamethoxy-7,8-dimethyl-7-dibenzo cyclooctenol	Biphenyl cyclooctene type	C_28_H_36_O_8_	500.58	(148)
6	Wuweizisu C	Biphenyl cyclooctene type	C_22_H_24_O_7_	400.42	(148)
7	1,2,13,14-tetramethoxydibenzo-cyclooctadiene-3,12-O-β-D diglucopyranoside	Biphenyl cyclooctene type	C_34_H_48_O_16_	712.74	(149)
8	Schilignan F	Tetrahydrofuran type	C_26_H_34_O_13_	554.20	(146)
9	19(R)-hydroxylwuwei-zidilactone H	Triterpenoid type	C_28_H_36_O_11_	548.58	(127)
10	Schisdilactone H	Triterpenoid type	C_30_H_38_O_11_	574.61	(150)
11	Schisanlactone I	Lanolin alkane type	C_32_H_50_O_5_	514.73	(30)
12	Schinalactone D	Cyclohexane type	C_32_H_50_O_5_	514.73	(30)
13	ganwuweizicacid	Cyclohexane type	C_30_H_46_O_3_	454.68	(151)
14	Propiniclactone A	Cyclohexane type	C_31_H_48_O_5_	500.71	(152)
15	Kadsudilactone C	Cyclohexane type	C_30_H_42_O_4_	466.65	(152)
16	Schisandronic acid	Cyclohexane type	C_30_H_46_O_3_	454.68	(153)
17	Henrischinin A	Cyclohexane type	C_30_H_42_O_5_	482.65	(153)
18	Icariside E4	Other types	C_26_H_34_O_10_	506.54	(146)

### 3.1 Lignans

Lignins are the most abundant and primary active metabolites in SCF (44). Lignans exist in free and glycoside forms in the seed coat and kernel. Eighty-six lignans have been identified, with the most active including schisandrol A, schisandrol B, Sch A, Sch B, and schisandrin C (Sch C) (45). Kosman et al. demonstrated the feasibility of extracting lignans from SCF seeds using fatty oils. Employing three distinct oils-corn, olive, and sunflower-for extraction, the researchers performed qualitative and quantitative analysis of five major *schisandra* lignans: Schisandrol A (≈ 0.1%), Schisandrol B (≈ 0.083%), Schisantherin A (≈ 0.022%), Schisandrin (≈ 0.018%), and Schisandrin B (≈ 0.09%). The total quantified lignan content reached approximately 0.3%. Notably, the lignan composition showed no significant variation across extracts obtained with different oils. Furthermore, the relative proportions of individual lignans exhibited a consistent ratio of approximately 1:1:0.2:0.2:1, which is reported as optimal for the adaptogenic properties of *schisandra*-based preparations (46). Sch A exhibits antioxidant, anti-inflammatory, anti-cancer, and liver-protective effects (47). Sch B has anti-inflammatory effects *in vivo and in vitro* (48, 49), regulates lipid metabolism (50), and provides liver protection (51). The levels of gomisin D, schisandrol B, kadsuranin, and kadlinolactone F in the roots are higher than in the fruits, and these metabolites are closely related to SCF’s antioxidant and anti-inflammatory activities (52, 53). Deoxyschisandrin is a bioactive benzocyclooctadiene lignan found in SCF. Using DS liposomes (DS-lipo) to enhance the liver targeting of 3 T3-L1 cells and inhibit adipocyte differentiation, it was found that DS-lipo suppresses lipid droplet accumulation, with a suppression rate of over 90% at 10 μM DS lipids (54). DS-lipo is thus a therapeutic option for lipid-related diseases and MASLD.

*Schisandra* lignans, valued for their broad-spectrum bioactivities, have been extensively investigated. Their pharmacokinetics (PK) and bioavailability critically influence therapeutic potential. Tissue distribution studies indicate preferential accumulation of schisandrin B and schisandrol B in hepatic and renal tissues (55, 56). The liver orchestrates lignan metabolism, with cytochrome P450 enzymes (CYP3A4 and CYP2B1) catalyzing biotransformation into multiple metabolites (57). Upon reaching hepatocytes, these metabolites mitigate OS by enhancing antioxidant enzyme activity, thereby exerting hepatoprotective effects. *Schisandra*-induced CYP activation may involve pregnane X receptor (PXR) agonism, with schisandrin A, schisandrin B, and gomisin B identified as primary ligands. Recent evidence confirms gomisin A activates PXR, augmenting bile acid metabolism/efflux while upregulating CYP3A expression in murine livers (58).

Lignans and metabolites undergo primary excretion via biliary, fecal, or urinary routes, typically exhibiting low cumulative excretion (59). Feng et al. (60) characterized schisantherin A metabolism through *in vitro* rat liver microsomes and *in vivo* (plasma/urine) studies. UPLC-Q-TOF-MS/MS analysis detected 56 urinary metabolites, 8 biliary, 19 plasma, and 5 microsomal, implicating both hepatic and extrahepatic biotransformation pathways. Another study also pointed out that schisandrin B is mainly excreted in the form of metabolites, with extremely low levels in urine, bile, and feces (61).

Notably, *Schisandra* lignan PK exhibits significant sex-related differences. Female rats demonstrate slower elimination rates and higher bioavailability versus males after single/multiple dosing. Schisandrin’s elimination half-life (t_1/2_) in females was 2-fold longer, while maximal plasma concentration (C_max_) was 10-fold higher than in males (62). The research results of Xu et al. are similar. They compared the pharmacokinetic differences between male and female rats after oral administration of 10 mg/kg pure schisandrin. It was found that the t_1/2_ time of schisandrin in female rats was prolonged by two times, and the C_max_ value was also increased by 20 times (63). This sexual dimorphism primarily stems from sex-dependent metabolism. CYP-mediated metabolism constitutes schisandrin’s primary clearance route (64), with CYP2B, CYP2C, CYP2D, and CYP3A crucially facilitating biotransformation (65). The activity of most CYP enzymes in female rats is significantly lower than that in male rats.

From a pharmacokinetic perspective, *Schisandra* lignans generally exhibit reasonable gastrointestinal absorption and favorable penetration into the liver-intestinal axis, supporting their potential for oral administration and application in hepatology. Despite certain limitations, their structural diversity and relatively high absorption rates position them as promising candidates for further drug development. Future research should prioritize strategies to enhance bioavailability and comprehensively explore their multifaceted bioactivities to fully realize their therapeutic potential.

### 3.2 Triterpenes

Among the 66 triterpenes discovered in SCF, 33 are cycloartanes, 31 are oleananes, and 2 are lanostanes (kadsuric acid and schisanlactone I). Recently discovered triterpenes from SCF stems include 24(*E*)-3α,12α-dihydroxyl-lanost-9(11),24-dien-26-oid acid and propinqtrilactones A and B (66, 67). The structural diversity of triterpenes endows them with antiviral, anti-inflammatory, hepatoprotective, and immunosuppressive activities. Regarding hepatoprotective activity, triterpenes ameliorate liver damage through intervening in OS responses (68, 69), making *Schisandra* plants and their active metabolites candidates for drugs against diseases such as fatty liver and viral hepatitis.

### 3.3 Volatile oils

Volatile oils are oil-like liquids that have a fragrant aroma and evaporate at room temperature. The main metabolites include β-caryophyllene, β-muscene, methyl butyrate, ethyl butyrate, and citral. *Schisandra* fruits, seeds, leaves, and vines all contain essential oils. The main metabolites of the essential oils in fruits and seeds are terpenes, with small amounts of alcohols, acids, and other oxygen-containing compounds. Those in the vines mainly consist of terpenes, lipids, and aromatics, while those in the leaves contain terpenes, alkanes, organic acids, alcohols, aldehydes, ketones, and aromatics (70, 71). These metabolites have antioxidant, anti-aging, pancreatic β-cell protective, and blood-sugar-lowering effects (72, 73).

### 3.4 Polysaccharides

*S. chinensis* polysaccharide (SCP) is a large molecule with a relatively high mass fraction up to 11.98% in SCF (74). The main metabolites include rhamnose, glucose, mannose, xylose, arabinose, and galactose (75, 76). Their bioactive effects include free-radical scavenging, reducing OS, inhibiting inflammatory factor production, anti-apoptosis, and liver protection (77). SCP can lower transaminase and blood lipid levels, alleviating OS damage to the liver (78), with especially significant effects against liver damage caused by high-fat diets (HFDs) (79).

### 3.5 Others

SCF also contains amino acids, organic acids, flavonoids, trace elements, and other minor components, including six essential amino acids for humans. Organic acids such as citric acid, malic acid, succinic acid, and protocatechuic acid have also been isolated from *Schisandra*, giving it a unique sour taste and contributing to its antioxidant, immunity-enhancing, gastric acid-regulating, and digestion-promoting properties (80). Flavonoids such as quercetin, myricetin, and kaempferol are present in the roots, stems, fruits, and especially leaves of *Schisandra*, where the concentration is nearly 50 times that found in the fruits. Finally, *Schisandra* contains small amounts of potassium, calcium, zinc, iron, and other elements, which also play roles in its pharmacological activity and health benefits.

## 4 Role of SCF in treating MASLD

The therapeutic efficacy of SCF and its constituent compounds against MASLD has been extensively characterized across diverse experimental models (see Table 2), which summarizes key pharmacological studies detailing the specific active metabolites, study models, dosage parameters, and the resulting mechanistic outcomes including regulation of lipid metabolism, antioxidant defense, and inflammatory responses.

**Table 2:** Summary of pharmacological studies of SCF in MASLD.

Natural products	Study model	Dose/concentration	Key findings	References
Schisandrin B	High fat/cholesterol/bile salts-induced hypercholesterolaemia mice	50–200 mg/kg for 6 days	Decreased hepatic TC and TG levels Increased hepatic indices	(50)
	D-gal sensitized mice	0.5–2 mmol/kg for 3 days	Enhanced hepatic mitochondrial glutathione redox status Increased Hsp70 level Prevented TNF-α-induced apoptosis	(51)
	HFD-induced NAFLD mice (*in vivo*)	50 mg/kg BW for 5 weeks	Decreased hepatic lipid content Activated autophagy through AMPK/mTOR pathway Promoted fatty acid oxidation	(87)
	FFA-stimulated HepG2 cells and primary hepatocytes (*in vitro*)	50 μM for 24 h	Activated autophagy through AMPK/mTOR pathway Decreased lipid droplet number	
	HSCs	2.5–5 μg/mL for 12 h	Reduced oxidative stress and Col-1/α-SMA expression Increased nuclear Nrf-2 and induced HO-1, NQO-1, GCLC	(119)
	C57BL/KSJ db/db mice	50 mg/kg for 2 weeks	Decreased body weight, blood glucose, serum insulin Improved insulin resistance and attenuated lipid deposition Inhibited Kupffer cells and inflammatory cytokines	(124)
	HFD-induced NAFLD mice	0.8 g/kg single dose; 50 mg/kg	Reduced hepatic palmitic acid and TG accumulation Decreased FAS and TNF-α expression Increased Nrf2 mRNA and HMG-CoA reductase activity	(125)
	FFA-induced steatosis in L-02 cells	1–100 μmol/L for 24 h	Alleviated lipid accumulation Decreased ADRP and SREBP-1 expression	(128)
	Male ICR mice	0.25–2 g/kg single dose	Increased serum TG, TC, Apo B48, NEFA, HGF Decreased serum VLDL and EAT mass	(130)
	AML-12 and RAW 264.7 cells	1–200 μM for 24–72 h	Induced G1 arrest and apoptosis via mitochondrial pathway Induced autophagy via PI3K/Akt/mTOR pathway	(132)
	Sch B- and SF oil-induced mice	0.125–2 g/kg single dose	Increased serum ALT, hepatic MDA and TG Decreased body weight	(131)
	AML12 hepatocytes	15 μM for 6 h	Activated MAPK and ERK1/2 signaling	(114)
Schisandrin A	HFD-induced NAFLD mice	40–80 mg/kg for 12 weeks	Inhibited weight gain; reduced ALT and AST levels Increased antioxidant enzymes (SOD) Decreased inflammatory cytokines (MDA, TNF-α, IL-1β, IL-6)	(154)
	HepG2 cells	30 μM for 48 h	Suppressed GSK3β signaling pathway	
	HFHC diet-induced NAFLD C57BL/6 J mice	0.5 g/kg for 15 weeks	Decreased plasma FFA and TG; increased HDL-C Enhanced hepatic β-oxidation and FA oxidation Increased fecal FFA/TG excretion	(112)
DS	HFD-induced obesity mice; MCD diet-induced NASH mice	100 mg/100 g diet for 6 weeks	Activated FXR/TGR5 signaling and leptin sensitivity Increased energy expenditure Reduced liver fat through central anorexia	(28)
	3 T3-L1 cells	10 μM DS-lipo and 30 μM DS for 6 days	Reduced cytoplasmic lipid droplet formation Increased liver targeting of liposome Inhibited adipocyte differentiation	(54)
GN	HFD-induced obese mice (*in vivo*)	2–10 mg/kg BW for 8 weeks	Downregulated lipogenesis genes Upregulated FA oxidation genes Inhibited hepatic steatosis	(85)
	HepG2 cells (*in vitro*)	10–100 μM for 24–48 h	Stimulated AMPK, ACC and SREBP-1c Inhibited LXR or PA-induced lipogenesis and TG accumulation	
	C57BL/6 mice (*in vivo*)	1–30 mg/kg BW for 4 d	Alleviated tunicamycin-induced hepatic ER stress and TG accumulation	(143)
	HepG2 cells (*in vitro*)	10–100 μM for 16 h	Inhibited ER stress and TG accumulation Decreased inflammatory and lipogenic gene expression	
Gomisin J	OA-induced HepG2 cells	10–40 μM for 24 h	Suppressed lipogenic enzymes and inflammatory mediators Increased PPARα expression Activated AMPK, LKB1, and CaMKII	(86)
SCF	3 T3-L1 cells (*in vitro*)	0, 20, or 150 μg/mL for 4–6 days	Inhibited adipocyte differentiation and lipid accumulation Reduced expression of C/EBPβ, C/EBPα and PPARγ	(90)
	HFD-induced obese rats (*in vivo*)	50 or 200 mg/kg BW/day for 5 weeks	Reduced body weight and blood lipid concentration Decreased epidermal and perirenal fat pad weight Increased HDL-cholesterol levels	
	Obese women	100 mL twice daily for 12 weeks	Decreased waist circumference, fat mass, fasting glucose Modulated gut microbiota	(135)
	HFD-induced NAFLD mice	100 mg/kg for 56 days	Reduced serum LDL-C and hepatic SOD levels	(79)
SCF extract	HFD-fed C57BL/6 mice and ob/ob mice (*in vivo*)	1 g/100 g diet for 6 weeks	Decreased serum LDL-C, ALT, fat mass, insulin and leptin Reduced inflammatory markers (TNF-α, MCP-1) Activated FXR signaling pathway	(27)
	HepG2 or 293 T cells (*in vitro*)	60–1,000 μg/mL for 24 h	Activated FXR signaling Increased SHP, PPARα expression Inhibited TG accumulation	
	t-BHP-induced oxidative hepatic damage in rats	300–1,200 mg/kg BW for 14 days	Increased GST, GCLC, GCLM levels Elevated SOD activity Reduced neutrophil infiltration and liver cell necrosis	(38)
	OA-induced HepG2 cells	200–400 μg/mL	Reduced acetylated lysine and H3K9 expression Inhibited HAT activity Reduced SREBP-1c protein expression	(99)
	HFD-induced NAFLD C57BL/6 mice	7.86–15.72 g/kg BW for 8 weeks	Decreased body weight, serum TG/LDL-C, liver TC/TG Increased antioxidant enzymes Modulated gut microbiota	(136)
	HepG2 cells (*in vitro*)	10–100 μg/mL for 6 h	Inhibited ER stress and intracellular TG Decreased inflammatory genes (IL-6, TNF-α, MCP-1) Reduced lipogenic genes (FAS, SCD1, ACC1)	(142)
	HFD-induced obese mice (*in vivo*)	100–300 mg/kg BW for 16 weeks	Inhibited ER stress and hepatic lipid accumulation	
	HCBD-fed mice	0.3–9.0% for 10 days	Increased HDL/LDL ratio and fecal TC Decreased hepatic TC/TG and serum TG Enhanced hepatic glucose-lowering effect	(93)
	HCBD-fed mice	1–9% for 10 days	Reduced serum and hepatic TG/TC levels Lowered hepatic glucose and ALT activity	(98)
	Sprague Dawley rats	0.45% FSE (1 mg/mL) for 8 weeks	Inhibited body weight, fat pad mass, liver weight Reduced liver lipid, TG, cholesterol and plasma lipid levels	(126)
	3 T3-L1 cells	0.5–5 μg/mL for 4 days	Increased glucose disposal rates Potentiated first-phase insulin secretion Activated PPAR-γ agonistic action	(104)
	Male Sprague–Dawley rats	200 mg/kg BW/day for 6 weeks	Enhanced hepatic insulin sensitivity Improved insulin signaling	
SCP	HFD-induced NAFLD mice	100 mg/kg for 6 weeks	Decreased serum AST, ALT, TG, TC, LDL-C Reduced hepatic FAS and ACC levels Increased HDL-C and UGP2/UGDH levels	(107)
	HFD-induced NAFLD mice	100 mg/kg for 12 weeks	Reduced liver index and hepatic TC/TG content Decreased serum TG, TC, LDL-C, ALT, AST Inhibited LXRα/SREBP-1c/FAS/ACC pathway	(97)
*Schisandra* fruit vinegar	HFD-induced mice	10 mL/kg for 6 weeks	Decreased body weight, liver weight, serum TG/TC/ALT/AST Increased SOD activity and HDL-C Upregulated PPAR-α, ACOX1, CPT1 expression	(118)
Wuzhi capsule	MCD diet-induced NAFLD mice	250 mg/kg for 5 weeks	Decreased liver TG, lipid droplets, serum ALT/IL-1β/IL-6 Increased PPAR-α, PPAR-γ, MCAD, LCAD expression	(122)

### 4.1 Regulation of lipid metabolism

Hepatic lipid metabolism dysregulation is a fundamental MASLD mechanism. The liver is crucial for lipid metabolism, responsible for coordinating the synthesis, output, and distribution of FAs. Excessive liver fat accumulation is the first key step in MASLD and a pathological characteristic of the disease. Imbalance in the synthesis and breakdown of lipids in cells, such as triglycerides (TGs), phospholipids, glycolipids, and cholesterol esters, leads to an excessive content of FAs in the liver, resulting in the deposition of lipids in the form of TGs within liver cells, which promotes the occurrence of MASLD (81). Among patients with hyperlipidemia, 2/3 of those with high TG levels and 1/3 of those with high cholesterol levels also have fatty liver (82), indicating that in this group of patients, there is an increase in fat synthesis and a slowdown in breakdown, leading to excessive fat accumulation. Therefore, restoring the lipid metabolic balance in the liver is key to treating MASLD.

Adenosine monophosphate-activated protein kinase (AMPK) is a highly conserved serine/threonine kinase with a broad impact on glucose metabolism. AMPK activation can be used on downstream target proteins to improve processes such as liver lipid metabolism, OS, and inflammatory responses, thereby alleviating MASLD (83). At the protein level, SCF extract significantly increases the phosphorylation of AMP-dependent protein kinase (pAMPK), reducing the activity of acetyl-CoA carboxylase (ACC), which increases FA oxidation levels and reduces fat deposition (84). Gomisin N (GN), a lignan derived from SCF, can restore AMPK phosphorylation, which ameliorates TG accumulation induced by liver X receptor (LXR) (85). GN participates in liver steatosis and adipocyte differentiation by activating the AMPK pathway, ameliorating HFD-induced liver steatosis. Gomisin J, another low-molecular-weight lignan from *Schisandra*, regulates lipogenesis and lipolysis by activating AMPK, LKB1, and Ca^(2+)^/calmodulin-dependent protein kinase II (CaMKII) in HepG2 cells, as well as inhibiting fetuin-A, thereby suppressing lipid accumulation (86). Sch B promotes lipid clearance, inhibits liver fibrosis, and increases FA oxidation by regulating the AMPK/mammalian target of rapamycin (mTOR) pathway to activate autophagy, thereby playing a role in the prevention or treatment of MASLD (87).

Peroxisome proliferator-activated receptors (PPARs) are important nuclear receptors, playing crucial roles in regulating processes such as liver fat synthesis, cholesterol metabolism, FA oxidation, and inflammatory responses, making them therapeutic targets for MASLD (88). The PPARγ subtype promotes lipogenesis by mediating the expression of lipogenic genes, such as FA synthase (FAS) and ACC 1, leading to intracellular TG accumulation (89). Extracts of SCF and its lignans inhibit the differentiation of 3 T3-L1 preadipocytes into adipocytes by downregulating PPARγ expression, thereby preventing lipid accumulation (90). The other subtype, PPARα, is primarily expressed in the liver, which is rich in FAs, and can induce the expression of apolipoproteins (APOs), regulating FA catabolism and reducing fatty degeneration (91). The livers of MASLD patients show a significant decrease in PPARα expression and activity, indicating abnormal lipid transport (92). The water extract of SCF can increase the expression levels of alanine aminotransferase (ALT) released from the liver after injury caused by the PPARα agonist fenofibrate (93), and it reduces liver TG, total cholesterol (TC), and glucose levels.

SREBP-1c is another important transcription factor involved in lipid metabolism, crucial in the formation and development of MASLD (94). When activated, SREBP-1c regulates the expression of genes such as FAS, ACC1, and stearoyl-CoA desaturase 1 (SCD1), inducing lipogenesis and excessive accumulation of fat in hepatocytes, thereby promoting the occurrence and progression of MASLD (95). Therefore, inhibiting the SREBP-1c signaling pathway reduces hepatic steatosis (96). SCPs significantly reduced the liver index, serum TG, TC, ALT, and aspartate aminotransferase (AST) levels in MASLD rats by 12.0, 31.3, 28.3, 20.1, and 15.5%, respectively. Serum high-density lipoprotein cholesterol increased by 26.9%, while liver TC and TG content decreased by 27.0 and 28.3%, respectively, alleviating hepatocyte steatosis and necrosis. In MASLD mice receiving SCP treatment, significant downregulation of liver lipogenesis genes, SREBP-1c, FAS, and ACC expression, and LXRα expression was observed, implying that the hepatoprotective effect of SCP in the MASLD mouse model is mediated by downregulating the LXRα/SREBP-1c/FAS/ACC and SREBP-2/HMGCR signaling pathways (97). Sun et al. (98) studied the effects of aqueous extracts of FSC pulp (AqFSC-P) on serum/liver lipid and glucose levels in mice fed a high-cholesterol/bile salt diet. AqFSC-P supplementation significantly reduced serum and liver TG levels (approximately 40%), total TC levels (27–46%), and liver glucose levels (13–30%), improving the fatty liver condition in mice fed hexachlorobutadiene. The mechanism of weight/fat and liver lipid reduction induced by FSC-P involves the inhibition of SREBP-1 and adipose differentiation-related protein expression. In the presence of *S. chinensis* berry ethanol extract (SCE), HepG2 cells treated with oleic acid (OA) showed a reduction in OA-induced lipid accumulation, mediated by a decrease in SREBP-1c expression (99).

### 4.2 Alleviating IR

IR is a central pathophysiological feature of MASLD that creates a vicious cycle of metabolic dysfunction. The mechanisms of lipid and glucose metabolism disorders in MASLD involve multiple factors, among which IR plays a key role, manifesting as reduced glucose utilization (100). Hepatic IR leads to increased liver glucose levels and TG accumulation by weakening insulin-mediated suppression of gluconeogenesis and regulating insulin-mediated TG metabolism, which triggers hyperglycemia and dyslipidemia. Therefore, alleviating hepatic IR is a therapeutic strategy for MASLD.

Glucose transporter 4 (GLUT4) plays an important role in glucose transport and metabolism, primarily expressed in insulin-sensitive tissues such as adipose tissue, myocardium, and skeletal muscle (101). Pathological damage or displacement of GLUT4 impairs glucose transport, ultimately inducing IR. The role of GLUT4 in cellular glucose uptake is regulated by AMPK (102). Jin et al. (103) purified a low-molecular-weight polysaccharide (SCPP11) from SCP and applied it to an insulin resistance model in Buffalo rat liver (BRL) cells. Their study found that SCPP11 upregulated GLUT-4 expression, thereby improving glucose consumption. This pharmacological effect of SCPP11 is associated with increased expression of the AMPK signaling pathway. When adenosine triphosphate (ATP) consumption and the AMP/ATP ratio increase, AMPK is activated under metabolic stress, enhancing glucose uptake and ameliorating IR. Hence, SCPP11 is a potential functional food ingredient for preventing and alleviating IR and fatty liver.

PPAR-γ participates in the development of IR through regulating adipocyte differentiation and modulating adipocytokine secretion. Activated PPAR-γ promotes glucose utilization and enhances insulin signal transduction, thereby counteracting IR. *Schisandra* fruit extracts rich in schisandrin, gomisin A, and angeloylgomisin H can agonize PPAR-γ to ameliorate hepatic IR (104). Insulin receptor substrate 1 (IRS-1) can be dephosphorylated by protein tyrosine phosphatase 1B (PTP1B), which blocks the insulin signaling pathway. Therefore, PTP1B inhibitors can enhance insulin sensitivity and glucose tolerance. The petroleum ether extract of *Schisandra* inhibits PTP1B activity, suggesting its potential as a functional food ingredient for the prevention and treatment of metabolic disorders (105).

Pyruvate is a central intermediate in the metabolism of carbohydrates, lipids, and various amino acids. In individuals with elevated hepatic TG levels, the flux of mitochondrial pyruvate carboxylase is increased (106). Pyruvate is primarily generated through dietary intake, and its production is influenced by acetyl phosphate (ACP) and nicotinic acid. In MASLD rats, the relative content of nicotinic acid significantly decreases, while that of ACP markedly increases, indicating abnormal pyruvate metabolism. After intervention with SCP in MASLD model rats, the relative levels of nicotinic acid were elevated, ACP levels were reduced, and serum d-glucuronic acid (d-GlcA) content increased, suggesting that SCP exerts hepatoprotective effects in MASLD rats by regulating pyruvate metabolism and d-GlcA-related metabolic pathways (107). Additionally, butyrate, a short-chain FA produced by microbial fermentation in the distal intestine and colon (108), influences the onset and progression of MASLD by inhibiting IR and attenuating hepatic mitochondrial OS (109). In MASLD rats, the relative expression of butyrate was low, but increased by SCP intervention, indicating that SCP may alleviate MASLD by modulating butyrate metabolism. The therapeutic effects of SCP in MASLD primarily involve metabolic pathways such as ascorbate and aldarate metabolism, pentose and glucuronate interconversions, nicotinate and nicotinamide metabolism, the tricarboxylic acid cycle, butyrate metabolism, and inositol phosphate metabolism. By detecting the expression of key targets in these metabolic pathways, including UDP-glucose pyrophosphorylase 2 (UGP2), UDP-Glc dehydrogenase (UGDH), ACC, and FAS proteins, the mechanism through which SCP exerts therapeutic effects in MASLD has been confirmed.

### 4.3 Antioxidation

OS plays a critical role in the progression from simple steatosis to steatohepatitis and fibrosis in MASLD. High levels of fat and cholesterol disrupt lipid metabolism balance, leading to mitochondrial dysfunction (110). During mitochondrial and peroxisomal FA oxidation, reactive oxygen species (ROS) are generated, triggering lipid peroxidation (111) and the development of obesity, hepatic steatosis, and even MASLD. Supplementation with Sch A alleviates HFD-induced MASLD by modulating hepatic lipid metabolism and OS. It significantly reduces hepatic free FAs (FFAs), lipid droplet accumulation, and the activity of enzymes involved in TG synthesis, while increasing the expression of genes related to hepatic β-oxidation and FA oxidation. Additionally, the expression of genes associated with cholesterol homeostasis and the fecal excretion of FFA and TGs are enhanced (112). Furthermore, Sch B protects against oxidative damage to liver tissue (113). One study elucidated the cytoprotective mechanism of Sch B-induced glutathione antioxidant response in hepatocytes. Sch B is metabolized by cytochrome P450 (CYP450), simultaneously generating ROS. The ROS activate the redox-sensitive ERK/Nrf2/ARE signaling cascade, leading to the expression of several glutathione-related enzymes. Reduced glutathione (GSH) and GSH-related antioxidant enzymes synergistically ameliorate OS (114). This antioxidant property of Sch B is a key mechanism delaying the progression of non-alcoholic steatohepatitis (NASH) and liver fibrosis. A study on the protective effects of six *Schisandra* lignans against liver injury found that these effects are partially related to the inhibition of cytochrome-mediated bioactivation. The hepatoprotective effects were most pronounced against oxidative damage in the liver, heart, and kidneys (115).

Additionally, the KEAP1-NRF2-HO-1 endogenous signaling pathway is crucial for reducing OS (116). Under normal physiological conditions, nuclear factor erythroid 2-related factor 2 (Nrf2) maintains low transcriptional activity. However, upon stimulation by ROS, kelch-like ECH-associated protein 1 (KEAP1) rapidly couples with Nrf2, activating the KEAP1/NRF2 pathway, which regulates the expression of antioxidant-related proteins and exerts antioxidant effects (117). A 6-week HFD can downregulate Nrf2 and heme oxygenase-1 (HO-1) and upregulate KEAP1 in rats. Yuan et al. (118) developed a unique *Schisandra* fruit vinegar (SV), distinct from grain vinegar and common fruit vinegars, derived from the fruit of SCF. SV downregulated KEAP1 expression while upregulating Nrf2 and its downstream targets HO-1 and SOD, thereby reducing the lipid peroxidation product malondialdehyde (MDA). Sch B inhibits hepatic stellate cell (HSC) proliferation and OS, with its protective effects involving the modulation of Nrf-2. It significantly enhances Nrf-2 activation and the expression of HO-1, NADPH quinine oxidoreductase (NQO1), and glutamate-cysteine ligase catalytic (119). These results suggest that SV enhances antioxidant capacity by modulating the KEAP1/NRF2/HO-1 pathway.

### 4.4 Anti-inflammatory response

Chronic inflammation is a hallmark of MASLD pathogenesis. Excessive lipid accumulation in the liver is closely associated with hepatocyte injury and Kupffer cell activation, which triggers inflammatory responses and induces the expression of interleukin-1 beta (IL-1β) and tumor necrosis factor-α (TNF-α) (120). PPARs play a role in inflammation by reducing the levels of cytokines such as interleukin 6 (IL-6), IL-1β, and TNF-α in liver tissues (121). The Wuzhi capsule, enriched with SCE, alleviates the “first hit” stage of MASLD by upregulating PPAR-α and PPAR-γ, thereby modulating hepatic lipid metabolism (122). A network pharmacology study identified Sch B, a major metabolite of Fuzheng Huayu formula, as directly binding to PPARγ, reducing the viability of HSCs (including T6 and LX-2 cells), and preventing liver fibrosis (123). Sch B protects against MASLD in db/db mice by reducing Kupffer cell accumulation in the liver, downregulating IL-1β and TNF-α infiltration, and suppressing inflammation (124). Kwan et al. (125) investigated the effects of Sch B on palmitic acid biosynthesis in liver samples from HFD-fed mice. Sch B treatment decreased FA synthase activity, hepatic TNF-α expression, and palmitic acid levels, which promote the progression of steatosis to steatohepatitis. Additionally, the treatment activated Nrf2, which mitigates the progression of NASH-related fibrosis. These findings suggest that Sch B has therapeutic potential for MASLD. Furthermore, the combined use of Fructus *Schisandrae* aqueous extract and atorvastatin reduced HFD-induced hepatic steatosis in Sprague Dawley rats, lowered liver ALT and AST levels, decreased macrophage infiltration (CD68), and reduced the tendency of calcium-induced membrane permeability transition in the liver (126).

The hepatoprotective active metabolites of SCF, such as lignans, triterpenes, volatile oils, and polysaccharides (127), have significant ameliorative effects on liver pathologies. Sch B, the primary active metabolite derived from *Schisandra* pulp, exhibits antioxidant, anti-endoplasmic reticulum stress (ERS), and anti-inflammatory activities in both *in vitro* cultured hepatocytes and *in vivo* rodent liver models. It inhibits FFA-induced steatosis in L02 hepatocytes in a dose-dependent manner, with mechanisms related to the suppression of lipogenesis and adipogenic differentiation (128). Long-term low-dose Sch B treatment (50 mg/kg/d for 14 days) reduced hepatic TG and TNF-α levels. However, a single high-dose injection of Sch B can elevate TC and TG levels in mice (129), with serum TG levels increasing by approximately 424%, promoting hepatic steatosis accompanied by hepatomegaly and mitochondrial damage (130). Similar findings on Sch B-induced hepatotoxicity were reported by Zhang et al. (131, 132), with mechanisms linked to increased serum ALT activity via PI3K/Akt/mTOR signaling. The safety and therapeutic efficacy of this treatment remain to be confirmed. Determining the optimal dosage and duration of Sch B treatment, with further experimental validation of the mechanisms underlying its effects during long-term low-dose therapy, is essential for maximizing efficacy and safety in humans.

### 4.5 Others

In addition to abnormalities in lipid metabolism, IR, OS, and inflammatory responses, SCF can intervene in MASLD through pathways such as GM modulation and ERS.

#### 4.5.1 Modulation of GM

The GM plays a role in the pathophysiology of metabolic diseases through the gut–liver axis, making the composition and alterations of the GM a critical factor in regulating the pathology of MASLD. When GM dysbiosis or intestinal barrier dysfunction occurs, bacteria and endotoxins enter the liver via the biliary tract and portal vein, activating hepatic cell surface receptors and releasing inflammatory factors, thereby damaging hepatocytes and ultimately leading to liver inflammation and fibrosis. Therefore, elucidating the interactions between the GM and liver-derived unique factors will provide new therapeutic targets for MASLD (133). The beneficial effects of SCF on metabolic diseases via influencing GM have been confirmed (134).

Song et al. (135) demonstrated that daily intake of SCF water extract (equivalent to 6.7 g of dried FSC daily) for 12 consecutive weeks modulated the composition of the GM, which is closely associated with changes in metabolic parameters such as fat mass, ALT, AST, HDL, and fasting blood glucose in obese women. SCF-treated subjects experienced improvements in obesity-related parameters including waist circumference, body weight, BMI, and fat mass. Cheng et al. (136) investigated the preventive effects of phenolic metabolites in *S. chinensis* bee pollen extract (SCPE) on MASLD and the modulation of GM in HFD-induced obese C57BL/6 mice. They identified 12 phenolic metabolites in SCPE, chiefly naringenin, rutin, and chrysin, that promote the growth, proliferation, or survival of probiotics, including *Lactobacillus* and *Bifidobacterium* strains, thereby exerting probiotic effects. Simultaneously, phenolic metabolites can inhibit the proliferation of certain pathogenic bacteria (137). SCPE modulates structural changes in the GM of obese mice, suppressing the growth of *Pseudomonas* and increasing the relative abundance of *Lactobacillus*, possibly attributable to the polyphenolic metabolites in SCPE. These findings suggest that polyphenol-rich SCPE, exhibiting high antioxidant activity, can alleviate HFD-induced obesity and metabolic syndrome, making it a candidate for the prevention of human obesity and MASLD.

#### 4.5.2 Relieving endoplasmic reticulum stress

ERS is an important mechanism in MASLD pathogenesis. The endoplasmic reticulum (ER) plays an important role in the folding and processing of newly secreted proteins and membrane proteins, the synthesis of lipids and sterols, and the storage of intracellular Ca^2+^. Stimulation, e.g., glucose deprivation, hypoxia, or OS, can inhibit the folding ability of the ER, leading to the accumulation of unfolded proteins and triggering ERS (138, 139). ERS can activate transcription of genes such as SREBP-1c and FAS, induce very-low-density lipoprotein expression, lead to lipid accumulation in liver cells, promote IR and inflammation, and exacerbate MASLD (140, 141).

Sch B reportedly ameliorates hepatic steatosis in cultured HepG2 liver cells and C57BL/6 mouse liver by resisting ERS. The stereoisomer GN of Sch B can ameliorate ERS-induced hepatic steatosis (142), reduce the expression of ERS markers and inflammatory genes—including glucose regulatory protein 78 (GRP78), C/EBP-homologous protein, and X-box binding protein-1 (XBP-1)—and reduce the accumulation of TGs. Administration of GN to HFD-induced obese *Drosophila melanogaster* resulted in weight loss, induction of specific upregulation of lipid storage droplets (Lsd)-2 and hormone sensitive lipase (Hsl), and downregulation of heat shock protein (Hsp90) family members (dGRP94), a key regulator of ERS response. These *in vivo* studies suggest that GN could prevent and treat obesity. The methanol extract of *S. chinensis* can significantly inhibit the streptomycin-induced accumulation of TG and the expression of ERS markers in HepG2 cells and mouse liver (143), preventing the development of MASLD.

**Figure 2:**
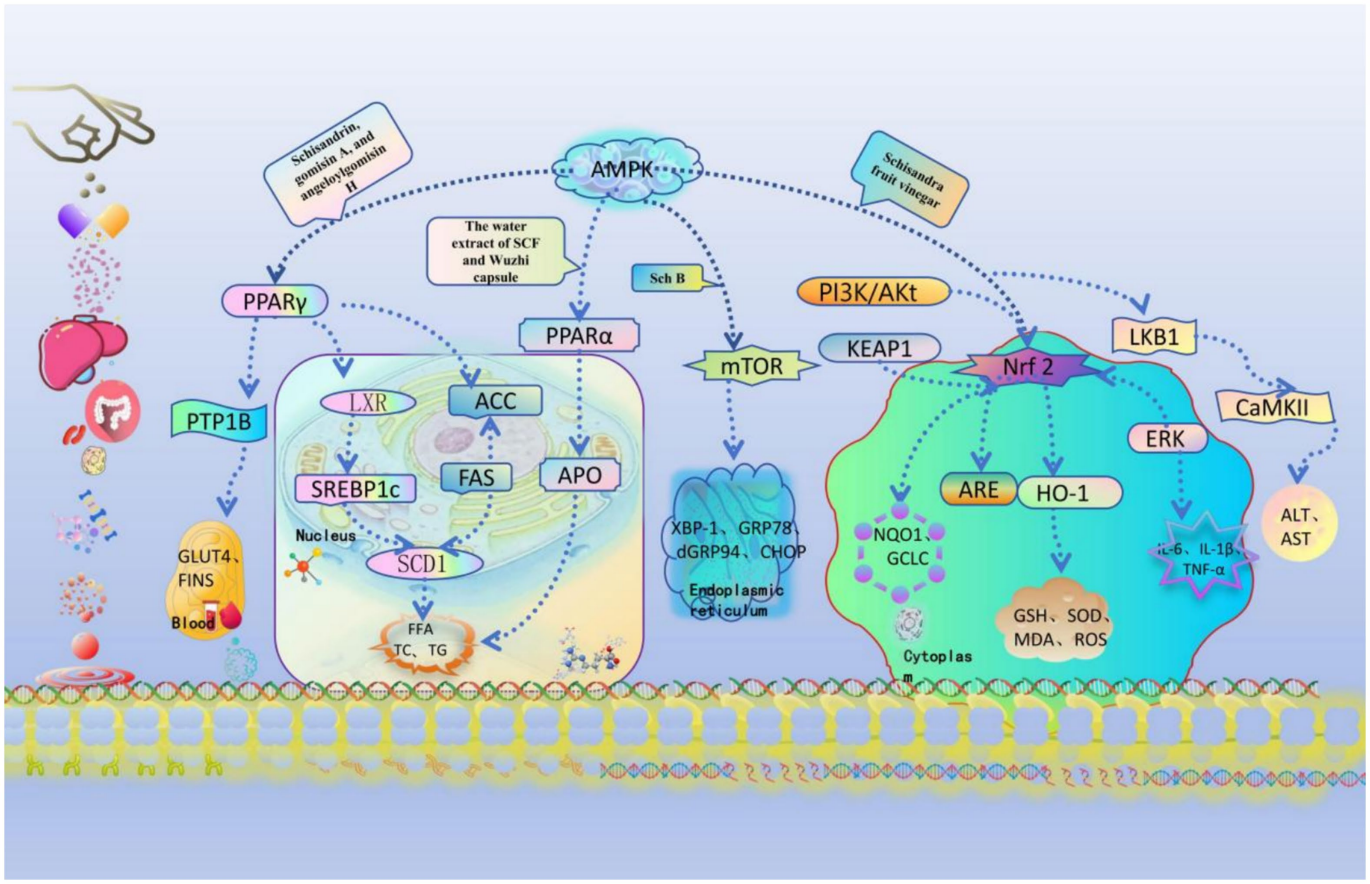
Potential therapeutic mechanisms and pathways of SCF in MASLD. SCF is involved in regulating the occurrence and alleviation of MASLD, mainly via lipid accumulation, insulin resistance, oxidative stress, inflammatory response, gut microbiota, and endoplasmic reticulum stress. The signaling pathways include induction of the activation of the AMPK pathway, up-regulation of PPAR-α to decrease LXR and ACC, down-regulation of PPAR-γ to promote insulin resistance and inhibit inflammatory response, suppression of Nrf2 to enhance HO-1 and SOD, antioxidant defense, autophagy, and potential biomarker therapy.

The multi-target therapeutic effects of SCF against MASLD are mediated through diverse molecular mechanisms (see Figure 2), which schematically illustrates the modulation of AMPK-dependent lipid metabolism, PPARα/γ signaling pathways, Nrf2/HO-1-mediated antioxidant responses, and PI3K/Akt/mTOR-regulated autophagy, alongside the inhibition of SREBP-1c-driven lipogenesis and endoplasmic reticulum stress.

## 5 Discussion

Our research focused on the therapeutic potential of SCF and its extracts in MASLD. During this investigation, we identified dose–response relationships as a significant concern requiring attention. *In vitro*, for purified lignans and extracts, effective concentrations range from 10 to 200 μM. Minimal active concentrations are consistently identified at 10–25 μM, with higher doses (50–100 μM) showing stronger inhibition of lipid accumulation and ER stress without significant cytotoxicity. *In vivo*, oral administration of SCF extracts or lignans at 50–300 mg/kg body weight is most common; high-dose groups (up to 1,200 mg/kg) are included to assess safety. Dose-dependent reductions in hepatic TG/TC, serum lipids, and liver injury markers are consistently observed, demonstrating therapeutic efficacy within this range.

Moreover, studies investigating long-term safety are highly limited. Most assess only short-term effects, typically over 4–16 weeks *in vivo*—a timeframe significantly shorter than the natural history of MASLD and insufficient to establish safety for chronic use. Data on chronic toxicity, such as high-dose hepatotoxicity or off-target effects, are rarely reported. Notably, several studies have documented hepatomegaly at elevated doses, including those by Pan et al. (50) and Zhang et al. (131). These investigations provide critical insights into the toxicity and potential risks associated with *Schisandra chinensis* metabolites, particularly under high-dose conditions.

Collectively, these studies demonstrate that Sch B and SCF exhibit dose-dependent toxicity, primarily manifesting as hepatomegaly, oxidative stress, and dysregulated lipid metabolism. The risks associated with Sch B are more pronounced, given its higher potency in inducing liver injury markers. Importantly, the observation by Pan et al. that hepatomegaly occurs alongside reduced hepatic lipids suggests that Sch B’s toxicity is not solely secondary to lipid accumulation but may involve distinct mechanisms. These findings underscore the need for caution when employing high doses of *Schisandra chinensis* metabolites for MASLD treatment. Clinically, establishing a safe therapeutic window is paramount, as the beneficial lipid-lowering effects of Sch B may be offset by hepatotoxicity at elevated doses. Consequently, further research is warranted to elucidate the underlying mechanisms and define dose limitations for safe long-term administration.

Given its central role in SCF-mediated hepatoprotection and quality control, we examined Sch B’s dual nature in fatty liver disease—therapeutic efficacy versus dose-dependent hepatotoxicity. High-dose Sch B induces hepatomegaly and lipid dysregulation (50), with outcomes dependent on three factors. Dosage shows a biphasic response: 50–200 mg/kg/day is hepatoprotective, whereas 0.8–2 g/kg causes hypertriglyceridemia and hepatomegaly (130). Efficacy is model-dependent—Sch B reduces lipids in obese/diabetic mice but may promote lipogenesis in normolipidemic mice at high doses. Duration matters: acute high-dose effects (24–72 h) are transient adaptive responses, whereas sub-chronic administration (2–5 weeks) permits compensatory homeostasis (87); thus, acute findings should not be extrapolated to chronic therapy. Accordingly, future research must avoid excessive doses, include fed/fasted states and adequate durations, define metabolic status, and avoid extrapolating hyperlipidemic model results to healthy populations. For clinical translation, a safe human equivalent Sch B dose offers a substantial safety margin versus animal toxic doses, though dose-ranging studies are needed. Sch B is more suitable for patients with metabolic dysfunction than for healthy individuals; clinical use requires regular liver/lipid monitoring and patient education against high doses or unsupervised concentrated extracts.

Furthermore, the pharmacological mechanisms of SCF against MASLD warrant critical examination. Our findings demonstrate that SCF and its extracts act through multiple interconnected mechanisms, including activation of AMPK and Nrf2/HO-1 pathways, suppression of cholesterol synthesis, enhancement of the GSH system, downregulation of ER stress markers, and modulation of gut microbiota. These mechanisms exhibit significant synergy: AMPK activation not only inhibits lipogenesis and inflammation but also enhances autophagy and antioxidant responses, while lignans modulate bile acid metabolism via FXR/TGR5 agonism, and polysaccharides preferentially target SREBP-mediated lipogenic programming—underscoring constituent-specific actions within SCF’s bioactive matrix. Despite this, most studies have focused narrowly on the AMPK/SREBP pathway, neglecting emerging targets such as TGR5 and GRP. Metabolite research has concentrated almost exclusively on lignans, with triterpenoids and volatile oils remaining underexplored. Moreover, systematic investigations into pharmacokinetic or pharmacodynamic interactions among different chemical classes within SCF are scarce, and direct comparisons of whole extracts versus individual constituents or defined combinations in clinical or preclinical trials remain limited. These collective gaps point to clear directions for future research.

Current research on SCF for fatty liver disease exhibits an overreliance on preclinical models. As summarized in Table 2, most studies utilize *in vitro* cellular models or rodent models. While these models capture key features of MASLD, such as lipid accumulation and inflammation, they fail to fully recapitulate the complexity of human MASLD, which involves interactions with the gut microbiota, genetic variations, and comorbidities. Clinical data, particularly from randomized controlled trials involving MASLD patients, remain scarce.

However, the broader challenge of modernizing TCM research also applies to SCF. The synergistic action of multiple constituents in SCF—such as lignans, polysaccharides, triterpenoids, and volatile oils—through interconnected pathways complicates the elucidation of which specific components are responsible for its therapeutic effects, a challenge not encountered with single-compound drugs. Standardization also remains a hurdle, as variations in cultivation, harvest, extraction, and geographic origin affect chemical profiles and reproducibility. Compared with other herbal medicines for MASLD—*Silybum marianum*, *Glycyrrhiza uralensis*, *Curcuma longa*, and *Panax ginseng*—SCF exhibits unique advantages. Its multi-component profile broadly targets lipid metabolism, IR, inflammation, and GM. Unlike silymarin, SCF lignans also modulate nuclear receptors and activate AMPK signaling. Compared to *Curcuma longa*, SCF shows comparable anti-inflammatory effects while better improving lipid profiles and insulin sensitivity. *Panax ginseng* enhances insulin sensitivity, but SCF has more pronounced effects on hepatic lipid metabolism, likely due to its unique lignan composition. Additionally, SCF benefits from dual food-medicine use with a long-established safety profile. However, unlike *Silybum marianum*, SCF lacks extensive clinical trial data and requires more rigorous studies to establish comparable evidence.

To address these challenges, several strategies are recommended. Omics technologies—including metabolomics, transcriptomics, and proteomics—should be integrated to systematically characterize the chemical composition and biological effects of SCF. Network pharmacology approaches can help decipher the complex interactions between multiple components and targets. Furthermore, quality control standards must be established, specifying minimum thresholds for marker compounds and implementing good agricultural and collection practices to ensure batch-to-batch consistency. Finally, well-designed clinical trials are essential to validate preclinical findings and establish evidence-based therapeutic protocols for SCF in MASLD management.

Several future research directions are suggested. First, current studies use extracts with varying concentrations of active components, and this heterogeneity hinders comparability. There is an urgent need to develop unified extraction protocols and determine minimum thresholds for key bioactive components to ensure consistency across studies. Standardization of extraction parameters—including solvent concentration, extraction duration, and purification protocols—coupled with quantitative profiling of critical bioactive constituents will enable reliable cross-study comparisons and accelerate translational research. Second, further investigations into the synergistic effects between lignans, polysaccharides, and minor components are warranted, as combined components often exhibit stronger efficacy than single compounds. Third, defining a safe therapeutic window is critical, and dose-ranging studies in humans are needed to balance efficacy and toxicity. Preclinical studies have highlighted potential hepatomegaly or lipid metabolic disturbances at high doses; thus, clinical trials should systematically evaluate dose-dependent efficacy and adverse events to establish optimal therapeutic doses. Fourth, existing studies have emphasized the roles of AMPK activation, FXR/TGR5 regulation, and antioxidant effects, but the crosstalk between these pathways remains insufficiently explored. Priority areas should also include the gut-liver axis and the interaction between autophagy and endoplasmic reticulum stress. Specifically, it is essential to elucidate how SCF modulates the gut microbiota and whether microbial metabolites such as short-chain FAs and bile acids mediate its therapeutic effects. Finally, qualitative and quantitative studies are necessary to identify the specific chemical metabolites responsible for the pharmacological activities of SCF. Modern biological approaches should be employed to clarify its bioactivity, pharmacological effects, and mechanisms/pathways at the molecular and genetic levels. Integrating metabolite profiling with the pharmacological evaluation of extracts will help establish reliable quality assessment criteria. This will facilitate the clinical application of SCF-based compound preparations, improve drug quality evaluation, and lay the foundation for developing functional foods and health supplements.

## 6 Conclusion

SCF, which has long been used in traditional medicine, attracts growing attention for its nutritional and medicinal value, with health products and compound preparations such as *Schisandra* tea, SCP oral liquids, and *Schisandra* tablets emerging. In recent years, advanced research technologies have spurred rapid progress in the extraction of active metabolites from SCF and the understanding of their biological activities and functions. This provides a foundation for harnessing the medicinal value of SCF and developing clinical applications of its bioactive metabolites. This article comprehensively summarizes the literature on the chemical metabolites and pharmacological effects of SCF. The diverse active metabolites of this medicine show impressive activity in alleviating MASLD, giving rise to a wide range of applications and high research value. With intensified research efforts, the potential of SCF in medicine and healthcare will be increasingly realized.

## Glossary

SCF: *Schisandra chinensis* Fructus
MASLD: Metabolic dysfunction-associated steatotic liver disease
TCM: Traditional Chinese medicine
IR: Insulin resistance
GM: Gut microbiota
T2D: Type 2 diabetes
BMI: Body-mass index
FXR: Farnesoid X receptor
FGF 15: Fibroblast growth factor 15
TGR5: Takeda G-protein-coupled receptor 5
GSK3β: Glycogen synthase kinase-3β
OS: Oxidative stress
Sch A/B/C: Schisandrin A/B/C
DS: Deoxyschisandrin
PXR: Pregnane X receptor
SCP: *S. chinensis* polysaccharide
HFD: High-fat diets
FA: Fatty acid
TGs: Triglycerides
AMPK: Adenosine monophosphate-activated protein kinase
ACC: Acetyl-CoA carboxylase
GN: Gomisin N
LXR: Liver X receptor
CaMKII: Calmodulin-dependent protein kinase II
mTOR: Mammalian target of rapamycin
PPARs: Peroxisome proliferator-activated receptors
APOs: Apolipoproteins
ALT: Alanine aminotransferase
SREBP-1c: Sterol regulatory element-binding protein 1c
TC: Total cholesterol
SCD1: Stearoyl-CoA desaturase 1
AST: Aspartate aminotransferase
OA: Oleic acid
GLUT4: Glucose transporter 4
ATP: Adenosine triphosphate
IRS-1: Insulin receptor substrate 1
ACP: Acetyl phosphate
PTP1B: Protein tyrosine phosphatase 1B
UGP2: UDP-glucose pyrophosphorylase 2
ROS: Reactive oxygen species
CYP450: Cytochrome P450
GSH: Glutathione
NASH: Non-alcoholic steatohepatitis
KEAP1: Kelch-like ECH-associated protein 1
HO-1: Heme oxygenase-1
HSC: Hepatic stellate cell
MDA: Malondialdehyde
TNF*-*α: Tumor necrosis factor-α
IL-1β/6: Interleukin-1 beta/6
ERS: Endoplasmic reticulum stress
GRP78: Glucose regulatory protein 78
